# Feasibility of Using New Sustainable Mineral Additions for the Manufacture of Eco-Cements

**DOI:** 10.3390/ma17040777

**Published:** 2024-02-06

**Authors:** S. Moreno, M. Rosales, J. Rosales, F. Agrela, J. L. Díaz-López

**Affiliations:** Construction Engineering Area, University of Córdoba, 14071 Córdoba, Spain; q12mosas@uco.es (S.M.); mrosales@uco.es (M.R.); jl.diaz@uco.es (J.L.D.-L.)

**Keywords:** olive biomass bottom ash, eucalyptus biomass bottom ash, mix recycled aggregates, recycled concrete aggregates, crushing treatment, sustainable mineral additions

## Abstract

Due to a continuously developing population, our consumption of one of the most widely used building materials, concrete, has increased. The production of concrete involves the use of cement whose production is one of the main sources of CO_2_ emissions; therefore, a challenge for today’s society is to move towards a circular economy and develop building materials with a reduced environmental footprint. This study evaluates the possibility of using new sustainable supplementary cementitious materials (SCMs) from waste such as recycled concrete aggregates (RCAs) and mixed recycled aggregates (MRAs) from construction and demolition waste, as well as bottom ash from olive biomass (BBA-OL) and eucalyptus biomass ash (BBA-EU) derived from the production of electricity. A micronisation pre-treatment was carried out by mechanical methods to achieve a suitable fineness and increase the SCMs’ specific surface area. Subsequently, an advanced characterisation of the new SCMs was carried out, and the acquired properties of the new cements manufactured with 25% cement substitution in the new SCMs were analysed in terms of pozzolanicity, mechanical behaviour, expansion and setting time tests. The results obtained demonstrate the feasibility of using these materials, which present a composition with potentially reactive hydraulic or pozzolanic elements, as well as the physical properties (fineness and grain size) that are ideal for SCMs. This implies the development of new eco-cements with suitable properties for possible use in the construction industry while reducing CO_2_ emissions and the industry’s carbon footprint.

## 1. Introduction

Concrete is the most widely used material for the construction of buildings and infrastructure worldwide [1], owing to its well-established excellent physical and mechanical properties [2,3,4], in addition to its capacity to be manufactured in various forms and sizes [5] which is advantageous in comparison to other construction materials. Due to this, advances in concrete construction techniques and reinforced concrete are closely tied to the economic development of modern societies.

Given the significance of concrete in developed societies, it is essential to discuss ordinary Portland cement (OPC), which has been the primary material for concrete production for the last 200 years [6].

However, when it is applied for concrete production, OPC involves the consumption of a substantial amount of resources, leading to the high consumption of natural resources and the production of CO_2_ emissions [7]. Approximately 5–8% of the world’s greenhouse gas emissions are attributed to the cement industry [8].

During the cement manufacturing process, CO_2_ is emitted in the following stages [9]: the calcination of limestone (CaCO_3_) to transform it into CO_2_ and CaO, which is responsible for 50% of its emissions; the burning of fossil fuels for clinker production (40% of CO_2_ emissions); and transportation and electrical consumption (the remaining 10%).

Furthermore, in recent years, various studies have shown that the production of one ton of OPC type CEM I 42.5 results in 845 kg of CO_2_ emissions, while OPC with additives type CEM II 42.5 produces 769 kg of CO_2_ [10].

As a result of the extensive use of concrete manufactured with OPC and the significant greenhouse gas emissions produced in cement manufacturing, the environment and people’s quality of life on the planet are being adversely affected. Therefore, numerous proposals for mitigating CO_2_ emissions related to the use of OPC have been the subject of research in recent years. These strategies encompass the utilisation of mineral additions, the development of cements derived from alternative clinkers, the implementation of carbon capture, utilisation and storage techniques, the adoption of alkali-activated cements and enhancements in both the efficiency of cement applications and structural solutions [11].

One of the most interesting methods to reduce CO_2_ is the addition of minerals, which, when incorporated into concrete in combination with Portland cement, contribute to the properties of both fresh and hardened concrete by enhancing its durability, strength and sustainability. These materials are known for their ability to reduce CO_2_ emissions because they significantly reduce the use of OPC [12].

Mineral additions to cement can be classified as inert fillers or supplementary cementitious materials (SCMs), depending on whether they exhibit pozzolanic or latent hydraulic reactivity [13]. The most commonly utilised SCMs include fly ash (FA), ground-granulated blast furnace slag (GGBFS), silica fume (SF) and calcined clays (metakaolin).

Presently, the primary mineral additions in cement production consist of GGBFS, FA and limestone filler. However, the extensive use of GGBFS coupled with advancements in steel-making technology have led to a decline in its availability, posing limitations for future CO_2_ reduction initiatives [12,14,15]. While FA is twice as abundant as GGBFS, its variable quality restricts its suitability to be used as a replacement, allowing only about one-third to be considered [15,16,17]. Limestone filler (LF), with a permissible inclusion in OPC of up to 15%, exceeds this limit and results in compromised strength and increased porosity values [18,19]. Silica fume, often favoured for its use in high-strength concrete, is characterised by its limited availability [16,20,21]. In contrast, calcined clays, identified for their abundance surpassing even that of cement, emerge as a promising alternative for cement replacement. They not only offer a comparable performance as a cement substitute but also present a sustainable solution due to their widespread availability [22,23].

Additionally, currently, new developments are underway, such as limestone calcined clay cement (LC3), which has emerged as a highly promising substitute for conventional cement, presenting a substantial reduction in carbon emissions within the cement industry. LC3, recognised as a low-carbon cement, is formulated by combining limestone with calcined clay subjected to high-temperature firing. The interaction between the alumina in the calcined clay and the carbonate in the limestone leads to the creation of a carboaluminate phase, imparting strength and durability to the cement [24,25].

Other studies in the field of cement additions are based on the production of self-repairing cementitious materials. A good example of this are self-healing bacterial-based cements, in which some species of bacteria are able to fill cracks in the cement and reduce its porosity [26]. The incorporation of bacterial substrates into concrete mixes and spores that will be activated after the appearance of cracks can contribute to the self-healing of concrete [27].

After examining both the conventional and novel SCMs currently employed in cement manufacturing, there are noticeable gaps in the exploration of new recycled materials derived from waste, such as mixed recycled aggregates (MRAs) from construction and demolition waste or biomass bottom ash (BBA) from biomass combustion.

Extensive evidence supports the notion that recycled aggregates (RAs), particularly recycled concrete aggregates (RCAs), from processed concrete waste have significant potential for use as aggregate construction materials, such as mortar and concrete. Various prior studies have affirmed that recycled materials can be effectively incorporated into concrete, even achieving substitution levels as high as 100%, albeit with a 10% reduction in compressive strength [28,29].

In contrast, there have been few experiments regarding the use of processed RAs (p-RAs) as SCMs. Asensio et al. [30,31] and Ge et al. [32] investigated the utilisation of construction and demolition waste (CDW) ceramic wastes (e.g., waste bricks, tiles or ceramic material) as alternative pozzolanic materials in cement production. These studies demonstrated that cements incorporating these materials yielded comparable results to those incorporating silica fume and fly ash [33,34].

As with RAs, BBA as a construction material has been extensively studied. Examples of this include its application in concrete manufacturing [35], mortars [36,37], road layers [38] and soil stabilisation [39]. Some studies have tested the use of BBA from multiple sources as a supplementary material to cement, such as ash from rice husks, bamboo, banana plants, corn cobs and palm oil [40].

Regarding BBA as SCMs, Carrasco et al. [41] examined the impact of replacing OPC with BBA in the production of building blocks, with substitution rates ranging from 10% to 90%. Their study concluded that such substitution is feasible, although some properties were adversely affected. This underscores the necessity for precise control over the properties of BBA. Medina et al. [42] investigated the impact of incorporating BBA as an SCM, substituting 10% and 20% of OP, with an analysis focusing on mechanical and durability properties. Their study concluded that the partial replacement of OPC with the examined BBA enables the preservation of properties akin to a conventional Class 42.5, Type II/A cement.

The aim of this study is to analyse the potential use of four recycled materials (olive biomass bottom ash (BBA-OL), eucalyptus biomass bottom ash (BBA-EU), mixed recycled aggregates (MRAs) and recycled concrete aggregates (RCAs)) derived from industrial waste as inert fillers or supplementary cementitious materials. To achieve this, the by-products are processed using mechanical means to achieve high fineness and specific surface area values. After obtaining the processed materials, their physical and chemical properties are evaluated. Subsequently, the mechanical and physical properties of the processed recycled materials are studied as inert fillers or SCMs in comparison to the addition of a conventional mineral. This study shows the feasibility of using these materials as SCMs, which implies a reduction in the percentage of applied cement by up to 25%, while maintaining their suitable properties and reducing the industry’s carbon footprint.

## 2. Properties of the Materials and Processing Systems Applied

### 2.1. Properties and Processing of Sustainable Materials

Three types of by-products were studied for their application as a mineral addition in the manufacture of eco-cement: BBA, MRA and RCA.

#### 2.1.1. Description of Sustainable Materials

BBA is a by-product generated from the combustion of forest pruning waste in a thermal treatment plant. It is the non-combustible part collected at the bottom of combustion furnaces. Two types of biomass bottom ash from two combustion plants were used in the present study.

(i.) Biomass bottom ash from biomass from the olive industry (BBA-OL). This type of ash was collected from the Puente Genil combustion plant in Cordoba, Southern Spain (37°26′58.6″ N 4°48′41.5″ W);
(ii.) Biomass bottom ash from eucalyptus forest pruning biomass (BBA-EU). Collected from the Ence treatment plant in Mérida, Western Spain (38°51′09.7″ N 6°21′32.9″ W).

Furthermore, two types of RAs from the Gecorsa company (the waste management plant in Cordoba, Spain; 37°49′09.1″ N 4°44′01.6″ W) were applied in this study.

(i.) An MRA refers to a granular material obtained from the recovery and processing of CDW with various types of stony materials, such as concrete, bricks, ceramics, asphalt and other stony elements;
(ii.) An RCA is obtained by crushing and processing waste concrete, such as fragments from structures, pavements, blocks or other disused concrete elements. These concrete waste materials are treated to remove impurities and then crushed to suitable sizes for use as aggregates in new construction projects.

#### 2.1.2. Processing of Sustainable Materials

**Figure 1:**
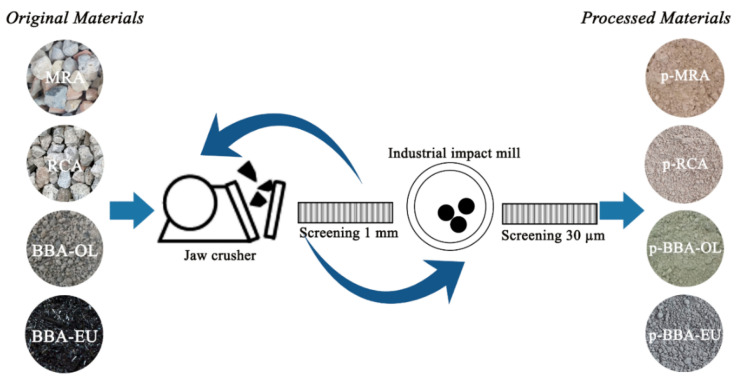
Material processing.

In this study, the material was processed to obtain a final product with a degree of fineness equivalent to that of cement (Figure 1).

The procedure was as follows:

(i.) Firstly, the materials to be processed (BBA-OL, BBA-EU, MRA and RCA) were introduced into a jaw crusher until the aggregate size was significantly reduced;
(ii.) Subsequently, the material was sieved through a 1 mm sieve, obtaining a material yield of approximately 60%;
(iii.) The above process was repeated with a material size greater than 1 mm. In this second screening phase, the yield of the pulverised material exceeded 80%.

Finally, the powdered material underwent micronisation in an industrial impact mill, from the manufacturer Bachiller, located in Barcelona, Spain, resulting in the production of the final products (p-BBA-OL, p-BBA-EU, p-MRA and p-RCA) with different particle sizes, the majority of which were less than 30 microns.

#### 2.1.3. Experimental Methods of Characterisation Properties and Results

**Figure 2:**
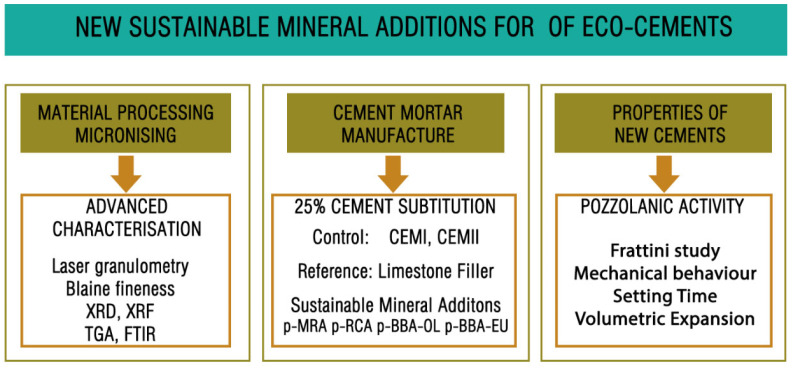
Experimental scheme of the study.

Figure 2 shows the methodology developed in this study. Once the material was obtained with a suitable particle size, it was subjected to advanced physicochemical characterisation, including tests of its particle size distribution, obtained via laser ray diffraction, XRD, XRF, TGA and FTIR, as well as measures of its density; sulphate, chloride, and organic matter content; and Blaine fineness.

Each of the mortars manufactured was subjected to a battery of tests to evaluate the possibility of applying the powdered by-products as a cement substitute and to compare them with the control and reference mortars. The tests conducted were the resistant activity index, the Frattini test (for pozzolanicity), the setting time and volumetric expansion.

##### Particle Size Distribution

This test entails measuring particle size distributions by analysing the angular variation in scattered light intensity when a laser beam traverses a sample. The angular scattering intensity data were subsequently analysed to calculate the size of the particles that contribute to the scattering pattern, employing the Mie theory of light scattering. The particle size is recorded as a sphere diameter equivalent to the volume.

**Figure 3:**
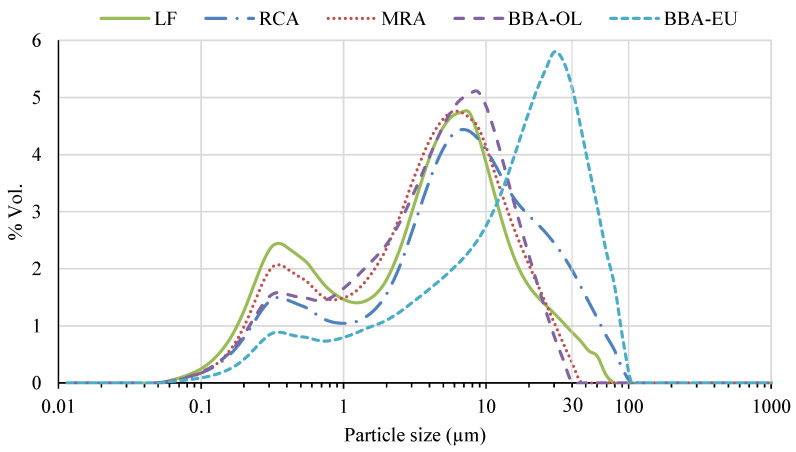
Grain size distribution of materials involved.

Figure 3 shows that p-BBA-EU presented a higher particle size than that of the other materials studied (p-BBA-OL, p-RMA and p-RCA). The physical properties, such as the Blaine fineness of the material (Table 1), showed a lower specific surface area in p-BBA-EU compared to the rest of the materials studied, which may be mainly due to the hardness of the material. Even so, this Blaine fineness value was remarkably close to the usual OPC values (2500–3500 cm^2^/g). Although BBA, regardless of its origin, has a high friability ratio [43], BBA-EU showed a higher amount of resistance in the crushing process and therefore a higher hardness value.

Table 1 supplies information on the different physical, chemical and mineralogical properties of the processed materials.

The results presented in Table 1 indicate that the particle size distribution of the various processed materials is variable. According to the R_30_ grain size distribution data (the proportion of particles in the powder with a grain size larger than 30 micrometres (µm)), the majority of the materials have a grain size much smaller than 30 µm, suggesting their suitability as cement substitute materials. This is because finer-grain particles provided a larger specific surface area (and consequently, a higher reactivity), a lower porosity value in the final material, and therefore a greater mechanical strength [44].

These findings were reinforced by the Blaine fineness data, in which, similar to the case of R30, a higher Blaine fineness value could accelerate the cement’s hydration rate, workability or pozzolanic reactivity [45].

To achieve this fineness, extensive pulverisation processes were conducted. However, the friability of the first materials meant that not all reached the same degree of fineness. In the case of BBA-EU, it showed a lower specific surface area compared to the rest of the studied materials, especially when compared to the results of BBA-OL, which achieved a higher degree of fineness due to its high friability.

##### Real Density

This test enables the determination of the volume of a sample comprising irregularly shaped particles. The density can be determined when the mass of the sample is known. The test, as stipulated in the UNE-EN 1097-7 standard, involves substituting a specific quantity of a liquid with a known density for the test sample. Within a pycnometer of the known volume, the test sample is introduced and completely immersed in the liquid. The volume of this liquid is computed by dividing the mass of the added liquid by its density. The volume of the test sample is then calculated by deducting this volume from the volume of the pycnometer.

The different densities of the materials in the table were around 2500 kg/m^3^, slightly lower than that of the conventional cement, which was around 3150 kg/m^3^. This fact showed that the mixes using these materials were somewhat more voluminous. Recycled materials must meet certain limits for these properties according to different standards. According to the most restrictive standard, the density of the recycled asphalt mixture (MRA) should be greater than 2200 kg/m^3^ [46], a requirement that was met by all the studied materials.

##### Sulphate, Chloride and Organic Matter Content

In addition to purely physical properties, the chemical composition of each material was evaluated, for which a study of the sulphate ion content was conducted via spectrophotometry in accordance with the UNE EN 1744-1 standard. The water-soluble sulphate content of the materials was expressed as a percentage of SO3. Additionally, a study of the water-soluble chloride content by the Volhard method (UNE-EN 1744-1) and a study of the organic matter content by the potassium permanganate method (NLT-118/91) were conducted.

The pozzolanic capacity of the materials is strongly determined by their chemical composition, especially the content of Si, Fe, Al and CaO, although the organic matter content and the crystalline structure of minerals are also factors influencing the pozzolanicity of the material [38,47]. In this case, it was observed that the amount of organic matter in BBA was higher than in the other materials due to its origin, which could negatively affect its pozzolanic capabilities [48]. However, its chemical composition was favourable, especially the amount of Al present in p-BBA-EU.

The content of soluble sulphates in acid and chlorine was higher in p-BBA-OL than in the rest of the analysed materials. This was possibly due to the use of phytosanitary products associated with olive cultivation that contain sulphur and chlorine [49]. Although the presence of these chemical species could negatively affect the cement structure, their quantities were not sufficient for this to occur.

##### X-ray Fluorescence and X-ray Diffraction

An X-ray fluorescence analysis (XRF) enables the simultaneous determination of multiple elements within diverse materials. One of its remarkable features is its rapidity, making it one of the most potent techniques for instrumental chemical analyses. This capability stems from its ability to qualitatively and/or quantitatively analyse various sample types (solids, liquids) without inducing any alterations, making it a non-destructive method.

X-Ray diffraction (XRD) is a non-destructive technique that helps the identification of different phases in a sample, as well as the structural and microstructural characterisation of solids. An X-ray diffraction analysis was conducted using a BRUKER Theta-Theta model D8 Advance X-ray diffractometer without a monochromator, employing a 2.2 kW Cu anode. The diffractogram of the crystalline powder was recorded within the range of 5° to 70°. The X-ray generator tube was run at a current of 30 mA and voltage of 40 kV, with a variable divergence slit set at 6 mm.

**Figure 4:**
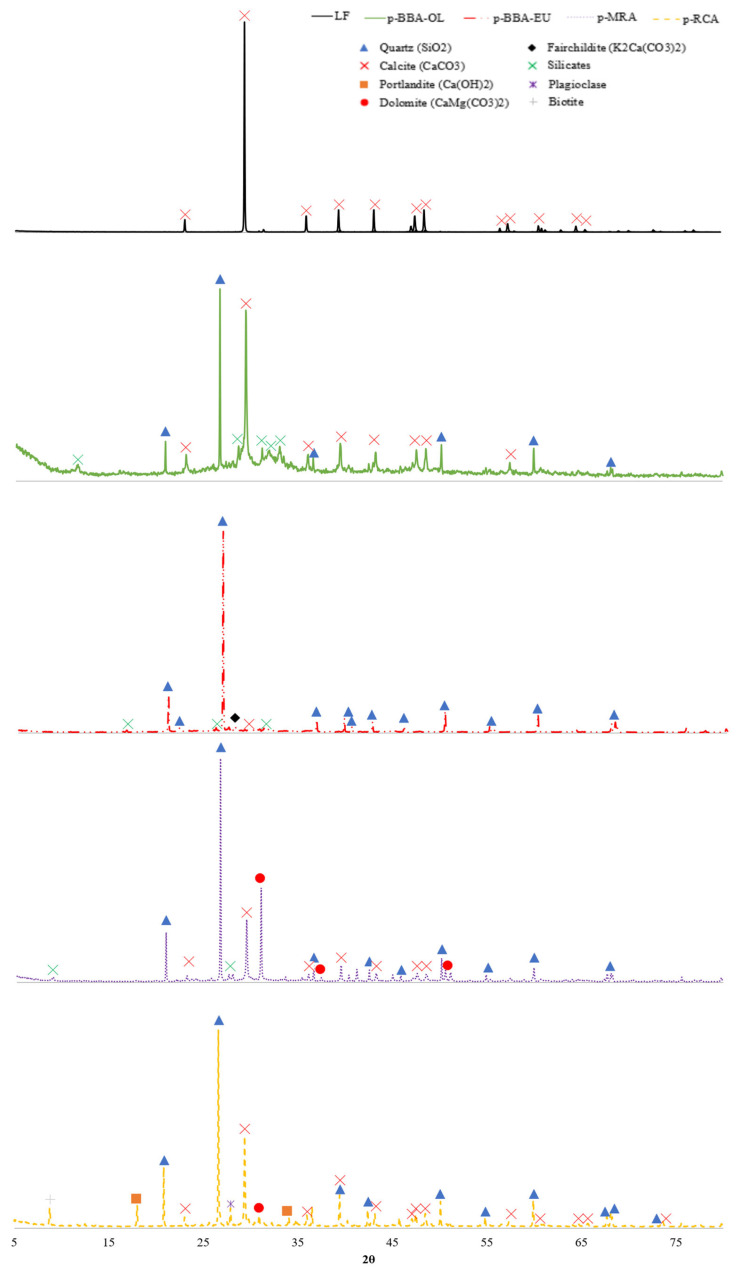
XRD of analysed materials.

The results obtained via XRF and XRD are shown in Table 1 and Figure 4, respectively.

Analysing the results shown in Table 1 and Figure 1, the following compositional and mineralogical characteristics were seen for each material:

The limestone filler showed a composition with 39% Ca, in addition to other elements with minor percentages. This implied a percentage of approximately 98% CaCO_3_ in the composition. This composition was consistent with the XRD analysis, in which only calcite was found, due to the high purity of the filler used.

The recycled aggregates, p-MRA and p-RCA, presented an elemental composition of calcium (15.7% and 18.7%), silicon (12% and 11.4%) and aluminium (2.49% and 2.21%). The p-RCA had a higher percentage of calcium than the p-MRA, due to the higher percentage by weight of natural aggregates, which generally originated from the same place as the limestone in the study area. This composition was consistent with previous authors [43,50].

However, the heterogeneity of the materials from CDW justified the variation in the ranges of elements found.

The similar composition found via XRF implied a similar mineralogy. Both of the RAs were mainly composed of quartzite and calcite. In addition, peaks of dolomite (due to the magnesium composition) and silicates, generally aluminosilicates derived from the ceramic material composing the RA, were found in the p-MRA. In the p-RCAs, portlandite was found, due to the presence of crushed concrete elements, and other minerals, such as plagioclase or biotite, due to the natural aggregates.

Biomass bottom ash was a highly heterogeneous material, and its composition was influenced by its origin, e.g., herbaceous material, wood, bark, etc.; in addition, the combustion technology had a significant influence [51].

In the present work, two typologies of biomass bottom ash were analysed, both with a majority composition, but with different percentages of calcium, silicon, aluminium, potassium, iron and magnesium. This composition was consistent with that found in the literature for various types of bottom ash using different biomasses and combustion technologies [52].

From a mineralogical point of view, the analysed biomass bottom ash was completely different. In the BBA-OL, mainly calcite and quartzite were found. The rest of the minerals found were different aluminosilicates and potassium silicates.

However, in the BBA-EU, quartzite was the most abundant mineral, as well as silicates, silicates and potassium carbonate.

##### Fourier Transformation Infrared Spectroscopy

**Figure 5:**
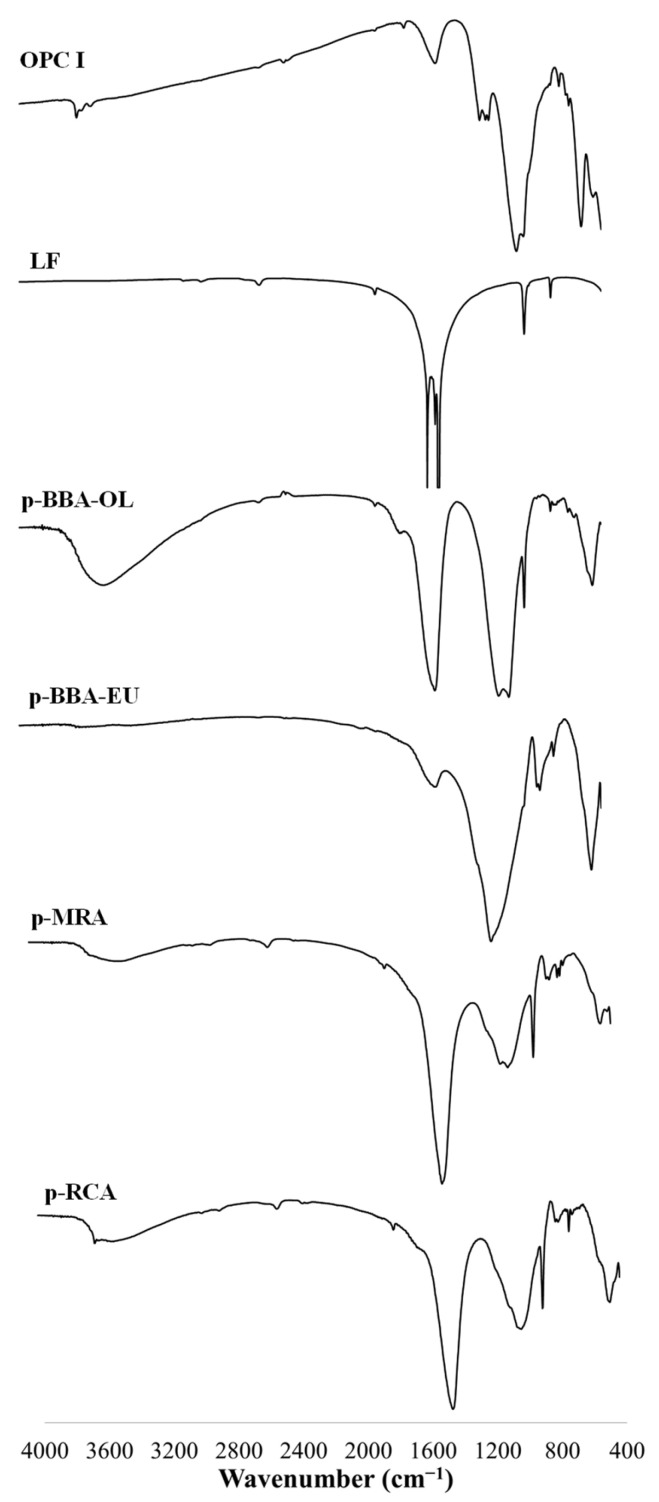
FTIR spectra of OPC I, LF, p-BBA-OL, p-BBA-EU, p-MRA and p-RCA.

The Fourier transform infrared spectroscopy (FTIR) test results reveal the absorption of infrared rays as they traverse the material. The absorption is analysed over a wavelength range from 4000 cm^−1^ to 400 cm^−1^. This test is instrumental in identifying the minerals constituting the sample, corroborating the results obtained through other mineralogy tests. Figure 5 displays the spectra absorbed by each material included in our research. To facilitate a thorough analysis, Table 2 quantifies the absorbance peaks at the specified wavelengths, aiding in the characterisation of the materials.

The peaks found around 3600 cm^−1^ were related to the presence of aluminosilicates, which were present in OPC I, p-BBA-OL, p-MRA and p-RCA [53]. For LF, p-MRA and p-RCA, there was a peak at 1435 cm^−1^, at 878 cm^−1^ and at 714 cm^−1^. This was due to the presence of calcite in the powder under study [54]. There were vibration frequencies at 1400 cm^−1^, 870 cm^−1^ and 710 cm^−1^ in LF, p-MRA and p-RCA. These bands were related to the presence of carbonates [55]. The peaks between 850 cm^−1^ and 880 cm^−1^ show that there were stretching bands in Si-O and the presence of -OH bending [56]. The asymmetric stretching of Si-O-Si was related to the peaks between 1000 cm^−1^ and 1060 cm^−1^ [57], which emerge in Figure 5 in the materials of p-BBA-OL, p-BBA-EU, p-MRA and p-RCA.

For the wavenumbers under 1000 cm^−1^, there was a peak in p-BBA-OL in 966 cm^−1^ that showed Si-O-K stretching due to the presence of K (Figure 4). For the Si-O stretching band found between 845 and 880, we found that OPC I, LF, p-BBA-OL, p-MRA and p-RCA showed peaks between these two values.

Materials like LF, p-MRA and p-RCA showed the asymmetric stretching of Si-O-Si or Si-O-Al around 714 cm^−1^. In the bands around 470 cm^−1^, it could be seen that p-BBA-OL, p-BBA-EU, p-MRA and p-RCA presented a peak in this frequency. This frequency was attributed to the presence of Si-O-Si or O-Si-O bending [58,59].

### 2.2. Properties of Conventional Materials

#### 2.2.1. Cements

For the development of this research paper, two conventional Portland cements were applied: CEM I 52.5R (OPC I) and CEM IV/A(V) 42.5R (CEM IV). In addition, a reference limestone filler (LF) was used.

It was observed that the Blaine fineness of the cement was similar to that in other previously published studies. With respect to the Blaine fineness of cements, other studies have shown values such as 4050 cm^2^/g [60], 3850 cm^2^/g [61] and 3800 cm^2^/g [62]. Other studies have also determined OPC densities similar to the results we obtained, with density values between 3036 kg/m^3^ [63] and 3110 kg/m^3^ [64].

#### 2.2.2. Limestone Filler

Betocarb filler by Omya Clariana (called LF) was used as an additional reference mineral during the development of our research.

Betocarb is a mineral filler made from calcium carbonate. It is known for its high purity and fine particle size, making it suitable for various applications in the construction industry. It is particularly recognised for enhancing important properties in materials such as cement and concrete.

It was observed that the LF was mainly composed of CaO, with a lower density than cement and a higher Blaine fineness (Table 3).

## 3. Experimental Methods and Results of Sustainable Cements

### 3.1. Mix Proportions

This section shows the dosages of the mortar mixtures developed with the recycled and powdered materials analysed.

The sand used for the manufacture of the mortars was CEN-NORMSAD standardised sand (SNS) in accordance with standard EN-196-1. In addition, the whole process of the manufacturing, curing and testing of the specimens was carried out in accordance with this standard.

To evaluate the pozzolanic capacity and strength of the new additions, each mortar mix was made by substituting 25% by weight of the cement with the new mineral additions.

Table 4 shows the nomenclature and properties of each manufactured mortar.

### 3.2. Test Procedures and Results of Mechanical Properties

#### 3.2.1. Frattini Test

Pozzolanicity is a crucial test conducted on cement substitutes to quantify the material’s capacity to fix calcium oxide. For this, the Frattini test is conducted, following the EN 196-5:2011 standard, on mixes prepared with the ratio of 75/25% (cement/sustainable mineral additions). In addition, the pozzolanicity of the two control cements and the reference mix manufactured with limestone filler was evaluated.

**Figure 6:**
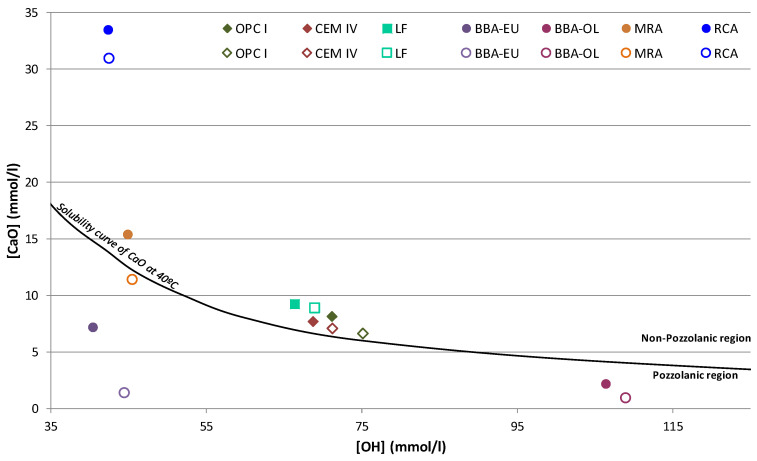
Findings of the pozzolanicity test at 8 days and 15 days for the mixes analysed.

The cements and mixtures were immersed in a saturated calcium oxide solution, and after 8 and 15 days, the amounts of hydroxyl ions and calcium oxide absorbed by the sample were measured. The values obtained are plotted on a graph in Figure 6, indicating whether the material is in the pozzolanic or non-pozzolanic region [65].

It was observed that the control cements and the reference lime filler blend were above the solubility curve showing the non-pozzolanicity of the blend. However, the BBA and p-MRA showed high pozzolanicity values after 15 days of the test, unlike the p-RCA, which resulted in a low pozzolanicity value in contrast to the results obtained by other authors [7]. The high pozzolanicity of BBA was due to the high presence of silica and alumina in the mineral additions (Table 1 and Figure 4), which were very high in p-BBA-EU and p-BBA-OL, directly showing the influence of their mineralogy and chemical composition on the pozzolanic capacity.

Previous studies have demonstrated the contribution of biomass ash to the internal microstructure of cement mortars; the silicates react with the cementitious compounds and form C-S-H gels, which improve the mechanical behaviour [66].

Regarding the p-MRA, it also met the 15-day pozzolanicity criterion (the point on the [CaO]/[OH-] graph below the CaO solubility isotherm at 40 °C); according to EN 197-1, to be considered a pozzolanic cement, this requirement must be met in all mixtures containing ≥11% pozzolanic additions. Previous studies have shown that a high percentage of ceramic particles in p-MRA leads to higher pozzolanicity values as the presence of calcined clay minerals such as kaolinite or dolomite (Figure 4) increases this property [31,67,68].

Another aspect to consider was the fineness of the material: a fine grinding of the material leads to improved pozzolanic properties and a higher spherification of the material particles, thus increasing the specific surface area and consequently the pozzolanic activity [69,70].

#### 3.2.2. Compressive and Flexural Strength

During the course of our research, an extensive study on the mechanical behaviour of mortar mixtures was conducted to assess the cementitious capacity of the materials under investigation. Following the UNE-EN 196-1 standard, mechanical tests were performed with a 25% cement substitution for each material. These tests encompassed short-, medium- and long-term evaluations.

The flexural strength was evaluated for two specimens, while the compressive strength was assessed for four specimens at three distinct ages: 7 days, 28 days and 90 days. The focus on long-term behaviour was paramount to comprehensively understand how these materials perform over extended periods.

There is research suggesting that the hardening capacity of certain materials, like BBA, increases significantly over the long term. It has been observed that in the long run, BBA’s hardening capacity can match or even surpass that of conventional cements in terms of its mechanical strength. This underscores the importance of evaluating the long-term behaviour of these materials for their potential applications and benefits in the construction industry [71,72,73]. Table 5 shows the results of the mechanical strength for compression and the flexural strength at 7, 28 and 90 days.

The control mixes showed normal values for the nature of the cements used. For the mixes with substituted cement, at 7 days, they showed strengths between 29 and 36 MPa for compression. The limestone filler was the worst compressive performer, with 29.74 MPa at 7 days, 33.86 MPa at 28 days and 36.27 MPa at 90 days. Among the bottom ash, the ash coming from eucalyptus showed a better mechanical behaviour than that from the burning of olive trees, showing 12% more resistance at 7 days, 12% at 28 days and 15% at 90 days for its compression as well as 4% at 7 days, 12% at 28 days and 17% at 90 days for its flexural strength. With respect the mortar mixes including the p-MRA and p-RCA as supplementary cementitious materials, the MRA powder showed a better mechanical behaviour than the RCA powder, although this difference was not very accentuated, being less than 2 MPa for the compression and less than 0.5% for the flexural strength at all the curing ages that were studied for these mortar mixtures.

**Figure 7:**
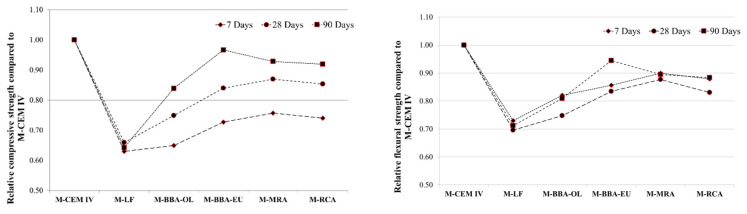
Relative compressive and flexural strength of the M-CEM IV mortar mix.

Figure 7 shows the relative mechanical strength of the M-CEM IV mortar mix.

The results shown in Figure 7 indicate that the M-BBA-EU mix achieved 97% of the compressive strength and 94% of the flexural strength with respect to the strengths of the M-CEM IV mixture with conventional cement. The RA showed good mechanical behaviour compared to the mix including CEM IV A(V) 42.5R at 28 days. The mixture developed with the MRA powder achieved 87% of the compressive strength and 88% of the flexural strength. Using the RCA powder as a supplementary cementitious material, the results were similar, reaching 85% and 83% of the compressive and flexural strength of the mixture including CEM IV A(V) 42.5R and conventional cement at 28 days, respectively. Other studies have found that, with mixes of 80% CEM I 52.5 and 20% MRA powder, their strengths at 28 days exceed those of conventional mixes with CEM II A/L 42.5 [43].

**Figure 8:**
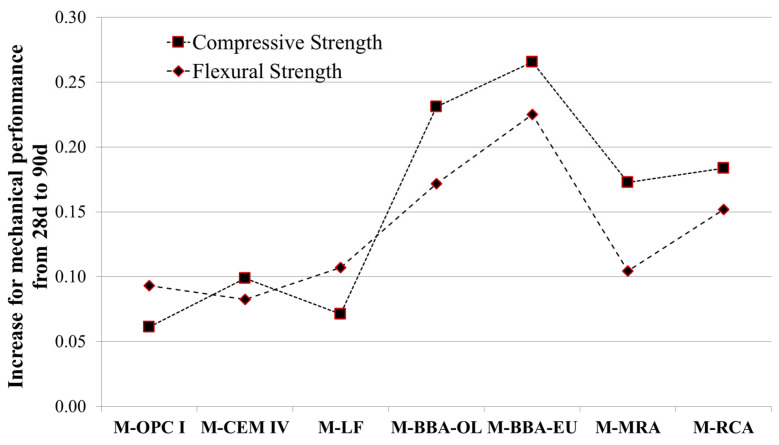
Strength increases between 28 days and 90 days for each mortar mix.

In order to show the high hardening provided in the long term, Figure 8 shows the increase in compressive and flexural strength presented by each of the mortar mixtures from 28 days to 90 days of curing.

The results show that the increase in the conventional mixtures (M-OPC I, M-CEM IV and M-LF) was between 6% and 10%. For the mortar mixtures including recycled aggregates (M-RCA and M-MRA), the increase in strength was between 10% and 18%. However, for the mortar mixtures including BBA (M-BBA-OL and M-BBA-EU), the increase was higher than the previous ones, ranging from 17% to 27%. This was due to the fact that BBA hardens in the long term by about 20% [74] or between 15% and 21% [43].

#### 3.2.3. Setting Time and Volumetric Expansion

The initial and final setting times were determined by measuring the loss of plasticity throughout the hardening process of the cement pastes using the Vicat apparatus.

The volumetric expansion during the hardening process, using Le Chatelier needles, was also determined. Both tests were conducted in accordance with the EN 196-3 standard.

**Figure 9:**
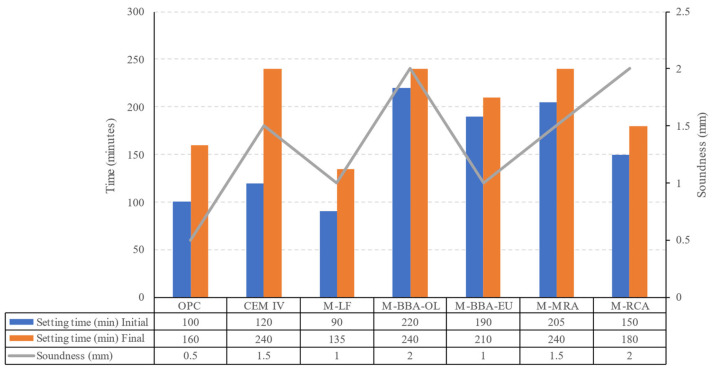
Setting time and soundness of blended cement.

Firstly, upon analysing the results presented in Figure 9, it was observed that all the samples exhibited values within the limits set by the EN 197-1 [75] for cements with resistance class 42.5 (the setting time test) or common cements (the soundness test). These limits included a maximum expansion of 10 mm and an initial time greater than 60 min.

For OPC, an initial time of 100 min, a final time of 160 min and an expansion of 0.50 mm were recorded. In the case of Type IV cement, there was an increase in both the initial and final times, reaching values of 120 and 240 min, respectively. Additionally, there was an increase in expansion up to 1.50 mm. These variations in the setting time and expansion values were attributed to the composition of the cement, which included a minimum percentage of 36% fly ash. The addition of fly ash has been extensively studied, including its effects on the setting time [76,77].

Regarding the M-LF mix, a slight increase in the expansion and a decrease in the setting time were indicated, reaching values of 90 min and 135 min for the initial and final times, respectively. This behaviour is well known and is attributed to the increased hydration process resulting from the addition of the limestone filler [78,79,80].

Analysing the effect of the byproducts evaluated as SCMs, the use of all materials resulted in an increase in both the initial and final setting times. M-pBBA-OL showed the most significant increment compared to OPC, ranging from 100 to 220 min for the initial setting time and from 160 to 240 min for the final setting time. On the other hand, M-pRCA showed the smallest increase compared to OPC, with 150 and 180 min for the initial and final setting times, respectively. Regarding the measured expansion, all pastes manufactured with by-products as SCMs indicated values similar to those of Type IV cement, ranging between 1 mm and 2 mm.

These increases in the setting time and slight expansions, attributed to the addition of biomass combustion ash [43,74,81,82,83] and CDW powdered material [43,84,85,86], have been reported in the literature and align with the results obtained in the laboratory in this study.

Velardo et al. [87] attributed this behaviour to various possible factors, including the reduction in the C_3_A phase percentage crucial in the early hours of hydration when the cement is replaced with these new SCMs. The more angular morphology and increased porosity of these additions compared to those of OPC particles impeded the binder movement, while the exothermic reaction between the cement and water, leading to the release of heat, water evaporation and eventual paste hardening, contributed to the observed effects. The reduction in cement quantity decreased the hydration heat, resulting in a delayed hardening of the pastes.

## 4. Conclusions

This study examined the feasibility of using four industrial byproducts processed as supplementary cementitious materials (SCMs) in the production of new sustainable cements. By completing this experimental campaign and analysing the results obtained, the following conclusions can be drawn:

- Although the R30 and Blaine fineness results indicated that the p-BBA-EU sample had a lower reactivity due to its smaller specific surface area, the pozzolanicity and mechanical strength results were positive. This suggests that a higher specific surface area for p-BBA-EU could enhance its cementitious properties.
- While great amounts of chlorine and sulphates are present in recycled supplementary cementitious materials, specifically in p-BBA-OL due to its agricultural origin, these values do not have a detrimental impact on the cementitious matrix or its potential use in reinforced concrete.
- The analysed by-products exhibited a less crystalline structure than the limestone filler. Additionally, minerals from the silicate group (in BBA-OL, BBA-EU and the MRA) and portlandite (in the RCA) phases were found. This mineralogy indicated the reactive potential of the analysed materials, translating into an enhancement of the properties of the developed cements.
- The evaluation of the pozzolanic capacity of each of the analysed by-products showed that p-BBA-EU and p-BBA-OL are materials with high pozzolanicity values. In the long term, it was observed that p-MRA also acquired a pozzolanic capacity. This property leads to the possibility of applying these by-products as SCMs, providing the resulting material with cementitious properties.
- The mortar mixture containing the additions of p-MRA and p-RCA is the one with the best mechanical behaviour at 28 days, reaching 85% of the resistance of the control mortar mixture.
- In the long term, the addition of p-BBA-EU shows greater hardening, reaching 95% of the long-term strength of the control mortar mix.
- Biomass bottom ash powder, regardless of its nature, shows a higher long-term hardening than MRA or RCA powder.
- The addition of the four analysed byproducts entailed an increase in both the initial and final setting times, along with a slight rise in soundness. However, all measured parameters adhere to the limits specified in EN 197-1 and do not negatively affect the developed sustainable cements. This implies their possible use as cementitious additives.

Based on this research, it can be concluded that both industrial by-products from processed biomass combustion and processed construction and demolition waste are suitable materials for the production of cements as supplementary cementitious materials. The characteristics of the processed by-products make them reactive materials that can outperform mineral additions like limestone filler and match the properties of a Type IV cement manufactured with coal fly ash.

## Figures and Tables

**Table 1:** Physical and chemical properties of sustainable materials.

Properties		p-BBA-OL	p-BBA-EU	p-MRA	p-RCA
Grain size distribution, R_30_ (µm) (%)		0.43	27.02	1.09	10.11
Real density (kg/m^3^)		2609	2331	2591	2402
Blaine fineness (cm^2^/g)		5080	2650	5600	4460
Organic matter content (%)		2.50	2.16	0.80	0.61
Acid-soluble sulphate (% SO_3_)		0.122	0.053	0.393	0.64
Chloride content (%)		1.196	0.323	0.414	0.139
Main components XRF (%)					
P		1.25	0.33	0.20	0.05
Si		10.5	22.0	12.0	11.40
Ca		19.6	6.36	15.7	18.7
Al		1.90	3.44	2.49	2.21
S		0.04	0.06	0.214	0.42
K		4.46	2.56	1.57	0.72
Mg		2.35	1.16	2.89	0.80
Na		0.191	0.73	0.31	0.23
Fe		1.33	3.07	1.42	1.28

**Table 2:** Principal infrared absorption bands.

Material	OPC I	LF	p-BBA-OL	p-BBA-EU	p-MRA	p-RCA
Wavenumbers (cm^−1)^	3642	1800	3466	1420	3600	3635
	1435	1434	1430	1052	1435	1796
	1150	1410	1040	783	1060	1437
	1100	876	966	691	1028	1000
	930	714	874	460	876	876
	880		460		775	783
	660				714	714
	527				460	467

**Table 3:** Physical and chemical properties of conventional materials.

Properties	OPC I	CEM IV	LF
Real Density (kg/m^3^)	3160	3010	2670
Blaine fineness (cm^2^/g)	4120	4370	6550
Chloride content (%)	0.03	0.09	0.007
Main components XRF (%)			
P_2_O_5_	0.15	0.43	0.02
SiO_2_	17.37	30.43	1.41
CaO	67.64	44.25	54.54
Al_2_O_3_	3.96	15.53	0.04
SO_3_	3.99	2.64	0.09
K_2_O	1.03	1.17	0.02
MgO	3.13	2.06	0.50
Na_2_O	0.32	0.38	0.28
Fe_2_O_3_	2.41	3.11	0.02

**Table 4:** Dosage of mortars (g).

Mixture		Dosages Serie (g)							
	SNS	OPC I	CEM IV	LF	p-BBA-OL	p-BBA-EU	p-MRA	p-RCA	Water
M-OPC I	1350	450	-	-	-	-	-	-	225
M-CEM IV	1350	-	450	-	-	-	-	-	225
M-LF	1350	337.5	-	112.5	-	-	-	-	225
M-BBA-OL	1350	337.5	-	-	112.5	-	-	-	225
M-BBA-EU	1350	337.5	-	-	-	112.5	-	-	225
M-MRA	1350	337.5	-	-	-	-	112.5	-	225
M-RCA	1350	337.5	-	-	-	-	-	112.5	225

**Table 5:** Mechanical strength of mortar mixes.

Mechanical Performance (MPa)	7 Days				28 Days				90 Days			
	Compressive		Flexural		Compressive		Flexural		Compressive		Flexural	
	µ	σ	µ	σ	µ	σ	µ	σ	µ	σ	µ	σ
M-OPC I	51.81	1.32	7.52	0.10	61.41	0.69	9.34	0.08	65.17	0.63	10.21	0.08
M-OPC II	47.21	1.11	7.18	0.17	51.36	0.87	8.72	0.10	56.43	0.51	9.44	0.06
M-LF	29.74	1.12	5.24	0.33	33.86	0.84	6.07	0.11	36.27	0.70	6.72	0.11
M-BBA-OL	30.67	1.03	5.89	0.21	38.46	0.69	6.52	0.09	47.35	0.52	7.64	0.11
M-BBA-EU	34.34	0.82	6.15	0.08	43.12	0.54	7.28	0.06	54.57	0.50	8.92	0.11
M-MRA	35.75	2.08	6.46	0.25	44.66	1.25	7.65	0.16	52.38	0.97	8.45	0.18
M-RCA	34.94	0.76	6.31	0.10	43.82	0.54	7.24	0.07	51.87	0.55	8.34	0.08

## Data Availability

The data is shown in the article.
